# NAT10 is critical to block RNA sensing-induced IFN-β transactivation in viral infection

**DOI:** 10.1128/mbio.01452-26

**Published:** 2026-08-04

**Authors:** Youngmin Park, Yan Liu, Jinshan He, Suha Eleya, Zhenyu Wu, Guillaume Nicolas Fiches, Dawei Zhou, Emily G. Watters, Zhixian M. He, Kateepe Arachchige Sudeera Nuwan Shanaka, Thurbu Tshering Lepcha, Jacob S. Yount, Netty G. Santoso, Jian Zhu

**Affiliations:** 1 Department of Pathology, College of Medicine, The Ohio State University 2647, Columbus, Ohio, USA; 2 Department of Microbiology, College of Arts and Sciences, The Ohio State University 2647, Columbus, Ohio, USA; 3 Department of Microbial Infection and Immunity, College of Medicine, The Ohio State University 2647, Columbus, Ohio, USA; University of Virginia, Charlottesville, Virginia, USA

**Keywords:** NAT10, Remodelin, interferon, immune signaling, IRF3, virus, antiviral

## Abstract

Cells detect invading viruses and produce type I interferons (IFNs) to stimulate an innate antiviral effector response. However, IFN levels must be fine-tuned to achieve antiviral efficacy while limiting hyperinflammatory and tissue-damaging effects. Here, we report that NAT10, a histone and cytidine acetyltransferase, regulates the production of type I IFNs and RNA virus infections. Depletion of NAT10 increased the expression of IFN-β and IFN-stimulated genes, and correspondingly impaired viral replication. Mechanistically, NAT10 dynamically associated with the IFN-β promoter and also negatively regulated IRF3’s chromatin associations through modulation of long noncoding RNAs that inhibit IRF3. Treatment of cells with Remodelin, a NAT10 inhibitor, similarly increased IFN-β expression and inhibited viral infections. Overall, our findings reveal NAT10 is a potential host-directed target for antiviral treatment via regulation of type I IFN.

**IMPORTANCE:**

Type I interferons (IFNs) signaling pathway is critical to cellular defense and innate immunity against evading pathogens, including viruses. However, induction of type I IFNs is fine-tuned to achieve the antiviral consequence while maintaining host cellular homeostasis. This paper presents a novel mechanism for the NAT10 protein to silence IFN-β induction through modulation of IRF3 activity at the promoter of IFN-β, and further demonstrates the therapeutic potential of the NAT10 inhibitor Remodelin to restrict viral infection while inducing IFN-β.

## INTRODUCTION

Type I interferon (IFN) signaling is an important component of host antiviral innate immunity and is induced upon recognition of pathogen-associated molecular patterns (PAMPs), including viral RNAs. In host cells, viral RNAs are detected by pattern recognition receptors (PRRs), such as retinoic acid-inducible gene I (RIG-I), triggering type I IFN induction (1). The signal is transduced to downstream adaptor proteins, mainly mitochondrial antiviral signaling protein (MAVS), which triggers its oligomerization and leads to activation of TANK-binding kinase 1 (TBK1) (1, 2). Activated TBK1 directly phosphorylates IFN regulatory factor 3 (IRF3) on specific serine/threonine sites (Ser386/396), which allows IRF3 dimerization, nuclear translocation to specific gene promoters, and transactivation of the genes, including IFN-β (3). Secreted IFN-β binds to receptors on cellular membranes and activates the Janus kinases that in turn activate signal transducer and activator of transcription (STAT) proteins, leading to the induction of hundreds of effector IFN-stimulated genes (ISGs), many of which have direct antiviral activities (3–5).

As mentioned, IRF3 is a crucial transcription factor required for induction of type I IFNs (3). Activated IRF3 binds to the IFN-β promoter and leads to its transactivation, which contributes significantly to the antiviral immune responses as well as the maintenance of cellular immune homeostasis (6). Multiple families of viruses have thus evolved mechanisms to antagonize IRF3 to evade the antiviral immune responses (2, 7, 8). The association of IRF3 with the IFN-β promoter recruits the histone acetyltransferase/transcription coactivator complex CBP/p300, which subsequently recruits the RNA polymerase II (Pol II) and activates transcriptional elongation (6, 9, 10). Such IRF3-mediated recruitment of RNA Pol II involves the chromatin remodeling achieved by CBP/p300-mediated BRG1/BRM-associated factor (BAF) complex of the SWI/SNF family, which permits the binding of TATA box-binding protein (TBP) to the promoter of IFN-β (6, 10).

N-acetyltransferase 10 (NAT10), a member of the GCN5-related N-acetyltransferase family, is an acetyltransferase that catalyzes histone (lysine acetylation) and RNA (cytidine ac4C) modifications. Earlier studies showed that NAT10 promotes replication of several viruses (11–15), while the underlying mechanisms of NAT10 remain to be fully elucidated. It is likely that NAT10 plays a general role in regulating antiviral innate immune responses, given that it has been reported that NAT10 regulates type I IFN signaling in a sepsis mouse model (16). We postulated that NAT10 may determine the outcome of viral infections through its functions that regulate immune responses showcased in other pathological conditions (17–22). The apparent non-specific proviral function of NAT10 is consistent with regulation of an antiviral pathway with broad effects. Our studies identified that NAT10 is a negative regulator of type I IFN signaling and directly suppresses the transactivation of IFN-β by interfering with IRF3 through modulation of IRF3-antagonizing lncRNAs. Overall, our results not only reveal a new role of NAT10 in dampening antiviral innate immune responses but also shed light on the potential for targeting NAT10 to augment antiviral immune responses using specific small-molecule inhibitors in the treatment of viral infections and associated diseases.

## RESULTS

### NAT10 promotes viral infection through suppression of ISGs

**Fig 1:**
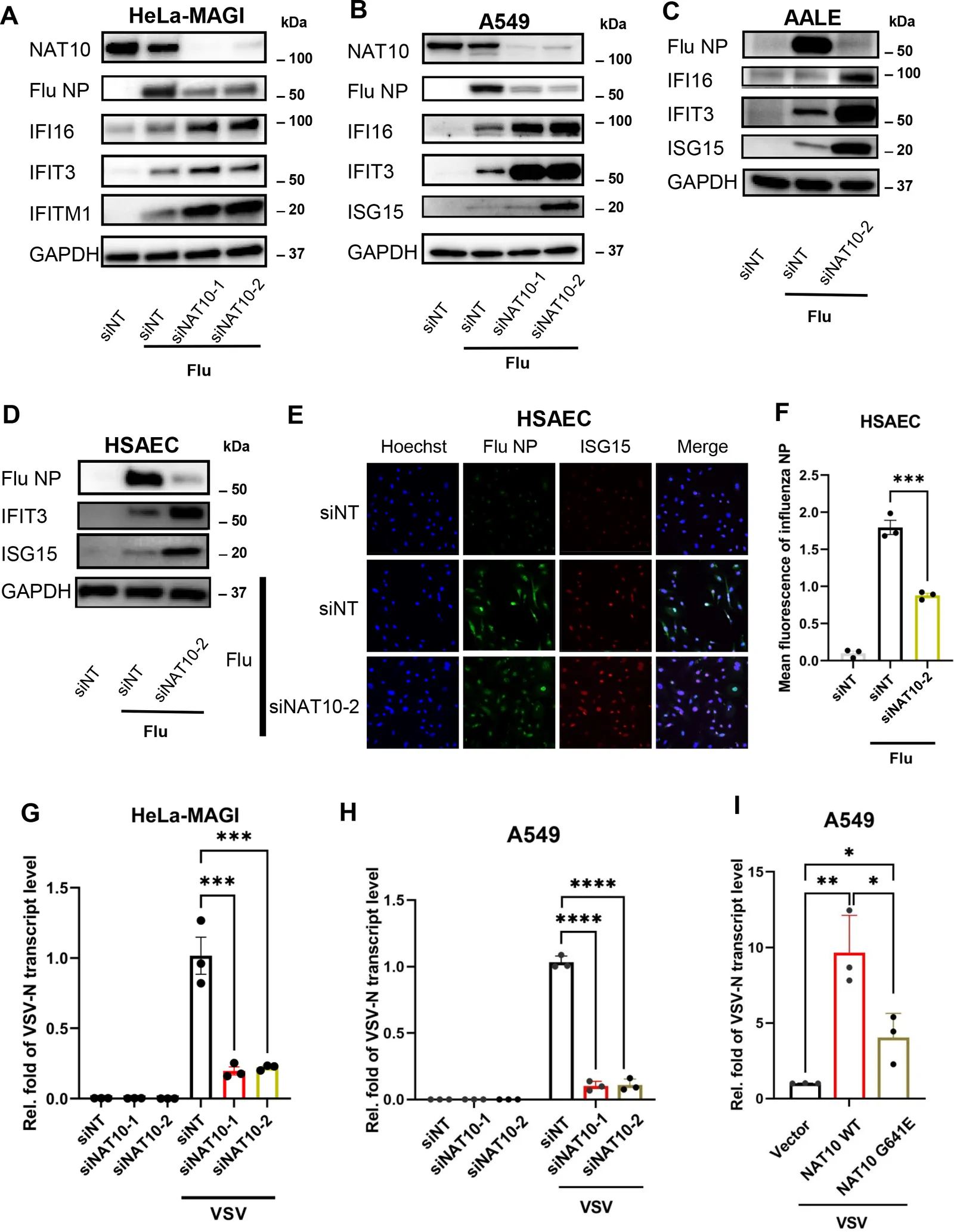
Depletion of NAT10 impaired viral infection while inducing expression of ISGs. (**A–D**) HeLa-MAGI (**A**), A549 (**B**), AALE (**C**), or HSAEC (**D**) cells were transfected with NAT10 or non-targeting siRNAs (siNAT10-1, siNAT10-2, siNT), followed by viral challenge with influenza. Expression of viral proteins (flu NP), NAT10, or ISGs was measured by protein immunoblotting using their specific antibodies. GAPDH was used as a loading control. (**E and F**) Protein expression of flu NP and ISG15 was visualized by dual-color immunofluorescence (Alexa 488: flu NP; Alexa 568: ISG15) in HSAEC cells transfected with the indicated siRNAs and infected with influenza (**E**). The mean fluorescence intensity (MFI) was quantified by using ImageJ (**F**). (**G and H**) HeLa-MAGI (**G**) or A549 (**H**) cells were transfected with the indicated siRNAs, followed by challenge with VSVs. Expression of viral gene (VSV-N) was measured by RT-qPCR. (**I**) A549 cells were transduced with lentiviral vectors expressing V5-tagged NAT10 WT or G641E, followed by VSV infection. Expression of viral gene (VSV-N) was measured by RT-qPCR. The results were presented as the Mean ± S.E.M of three biological replicates; *P  <  0.05, **P < 0.01, ***P < 0.001, and ****P < 0.0001, ANOVA and Bonferroni multiple comparison test for panels **G–I** or two-tailed Student’s *t*-test for panel **F**.

Human cervical epithelial cells, HeLa-MAGI, which are engineered from HeLa for overexpression of CD4 and LTR-β-gal, and human alveolar basal epithelial cells A549 were used in our study, as both of these cell lines have intact antiviral immune responses and are commonly used for host-virus interaction studies (23). To determine the impact of NAT10 on viral infection, NAT10 was depleted through RNAi-mediated gene knockdown (KD) in HeLa-MAGI and A549, challenged with influenza A virus (IAV). The siRNAs targeting NAT10 (siNAT10-1 and siNAT10-2) efficiently depleted endogenous NAT10 protein expression in HeLa-MAGI and A549, and caused a significant reduction in viral gene expression, measured by protein levels for IAV NP (Fig. 1A and B). The effect of NAT10 depletion on cell viability of HeLa-MAGI and A549 was negligible, as in previous results (Fig. S1A and B) (24). Of note, NAT10 depletion promoted virus-induced expression of ISGs in both cell types (Fig. 1A and B). We further confirmed that NAT10 depletion limited IAV protein production while increasing ISG levels in immortalized tracheobronchial epithelial cells (AALE) as well as in primary human small airway epithelial cells (HSAECs) (Fig. 1C and D; Fig. S1C). To further validate this, we used protein immunofluorescence to visualize the impact of NAT10 depletion on IAV NP and ISG15 expression during infection of HSAEC. Imaging showed that the NAT10 KD decreases IAV NP while increasing ISG15 (Fig. 1E and F). To assess whether NAT10 depletion also affects other viruses, we examined vesicular stomatitis virus (VSV) infection in NAT10-depleted HeLa-MAGI and A549 cells. Consistent with the IAV results, NAT10 knockdown significantly reduced VSV infection, as determined by VSV N transcript levels (Fig. 1G and H). In addition, overexpression of wild-type (WT) NAT10 significantly promoted VSV infection, while its acetyltransferase activity-deficient mutant G641E exerted much weaker effects (Fig. 1I; Fig. S1D), which further confirmed that NAT10 promotes VSV infection through its acetyltransferase activity. Overall, our results showed that the presence of NAT10 increases viral infection while suppressing induction of ISGs.

### NAT10 blocks ISG mRNA expression induced by the dsRNA analog poly(I:C)

**Fig 2:**
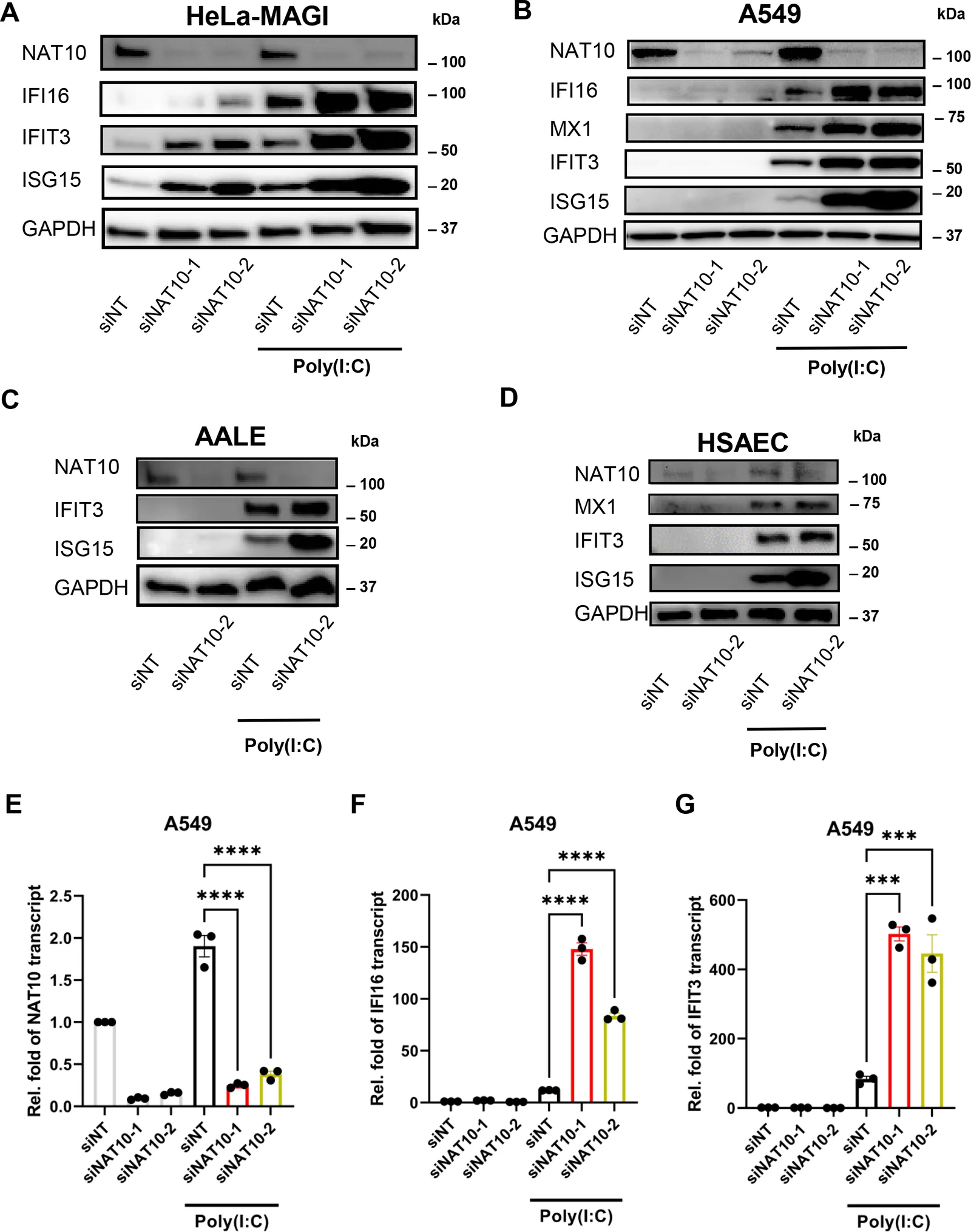
Depletion of NAT10 enhanced the poly(I:C)-induced ISG expression. (**A–D**) HeLa-MAGI (**A**), A549 (**B**), AALE (**C**), or HSAEC (**D**) cells were transfected with NAT10 or non-targeting siRNAs (siNAT10-1, siNAT10-2, and siNT), followed by poly(I:C) treatment (1 μg/mL). Expression of NAT10 or ISGs was measured by protein immunoblotting using their specific antibodies. GAPDH was used as a loading control. (**E–G**) A549 cells were transfected with indicated siRNAs, followed by poly(I:C) treatment. Expression of NAT10 (**E**), IFI16 (**F**), or IFIT3 (**G**) was measured by RT-qPCR. The results were presented as the Mean ± SEM of at least three biological replicates; ***P < 0.001, ****P < 0.0001, ANOVA and Bonferroni multiple comparison test for panels **E–G**.

To further investigate the potential mechanisms for NAT10 to regulate ISG expression, we alternatively stimulated cells with the synthetic double-stranded (ds) RNA analog, polyinosinic-polycytidylic acid [poly(I:C)] at optimized doses that activate viral RNA sensing and induce antiviral IFN signaling. As controls, we found that poly(I:C) treatment has no effect on NAT10 mRNA and protein levels or its cellular localization (Fig. S2A through C). We observed that NAT10 KD by siRNAs enhances poly(I:C)-induced ISG expression in HeLa-MAGI and A549 (Fig. 2A and B), and in the immortalized and primary human lung epithelial cells, AALE and HSAEC (Fig. 2C and D). These results demonstrate that poly(I:C)-induced ISG responses are decreased by NAT10 similarly to results obtained during RNA virus infection. Notably, ISGs were induced by NAT10 depletion even without poly(I:C) treatment in HeLa-MAGI but not in A549 (Fig. 2A and B), indicating that NAT10 could play a role in suppressing the basal IFN production in certain cellular microenvironments, which is consistent with a recent report and may be related to its R-loop regulatory activity (25). To study whether NAT10 plays a direct role in regulating type I interferon induction, we primarily employed A549 as the cell model for the following experiments since it provided a more quiescent background of IFN expression without the poly(I:C) treatment (Fig. 2B). Next, we sought to study whether NAT10 regulates ISG expression at the transcriptional level or not. Therefore, we further quantified the mRNAs of poly(I:C)-induced ISGs by RT-qPCR with or without NAT10 depletion. Our results showed that NAT10 KD significantly increases mRNA levels of ISGs (IFI16 and IFIT3) in poly(I:C)-treated A549 cells (Fig. 2E through G). Thus, we concluded that NAT10 impairs ISG expression at the transcript level.

### NAT10 suppresses poly(I:C)-induced IFN-β production in multiple cell types

**Fig 3:**
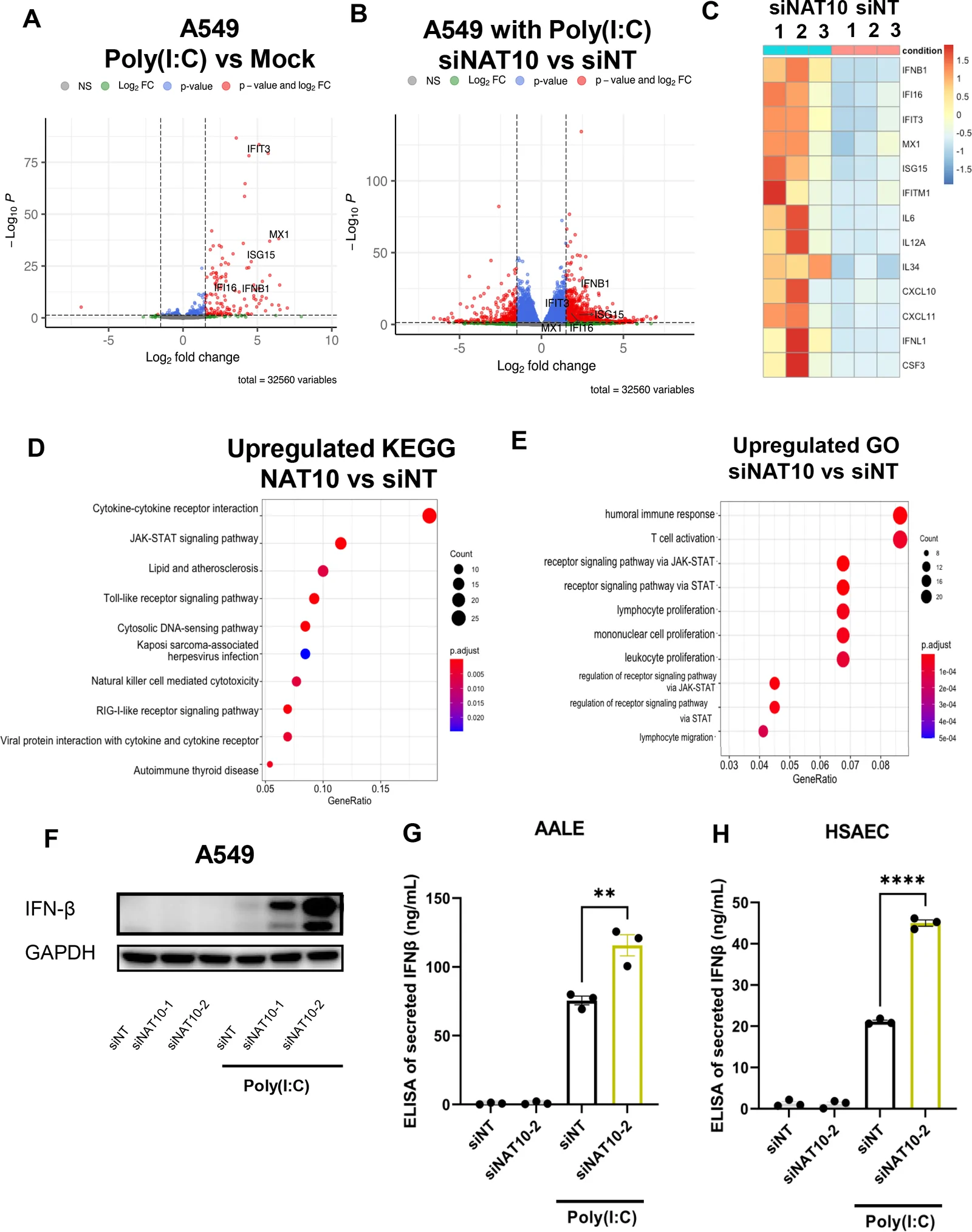
NAT10 suppressed RNA sensing-induced transcriptional activation of IFN-β. (**A–E**) A549 cells were transfected with NAT10 or non-targeting siRNAs (1:1 mixture of siNAT10-1 and siNAT10-2, siNT), followed by poly(I:C) treatment (1 μg/mL). Cell samples (triplicate 1–3) were prepared and processed for transcriptomic analysis by RNA-seq. Volcano plot of differentially expressed genes (DEGs) induced by poly(I:C) treatment (**A**), or NAT10 depletion (**B**) in A549 cells treated with poly(I:C). Heatmap of the upregulated DEGs due to NAT10 depletion (**C**). KEGG (**D**) and GO (**E**) analysis of the top 10 enriched pathways of upregulated DEGs due to NAT10 depletion. (**F**) A549 cells were transfected with NAT10 or non-targeting siRNAs (siNAT10-1, siNAT10-2, and siNT), followed by poly(I:C) treatment (1 μg/mL). IFN-β expression was measured by protein immunoblotting. (**G and H**) Supernatants of AALE (**G**) or HSAEC (**H**) cells transfected with indicated siRNAs and treated with poly(I:C) were collected for quantification of secreted IFN-β protein by ELISA. The results were presented as the Mean ± SEM of at least three biological replicates; **P < 0.01, ****P < 0.0001, two-way ANOVA for panels **G and H**.

To better understand the role of NAT10 in controlling antiviral immune responses, we performed RNA-seq analysis to characterize transcriptome alterations when NAT10 is depleted in A549 cells with poly(I:C) stimulation. First, our results showed that, as expected, poly(I:C) treatment induces the upregulation of type I IFNs and ISGs (Fig. 3A). IFN-β is among the first type I IFNs produced in most non-immune cell types, such as epithelial cells (26–28), and its expression in A549 cells was prominently upregulated upon poly(I:C) treatment. Enrichment of poly(I:C)-induced type I IFN pathway and other relevant pathways were confirmed by Kyoto Encyclopedia of Genes and Genomes (KEGG) and gene ontology (GO) analyses (Fig. S3A and B). Next, we compared the transcriptomic profiles in poly(I:C)-stimulated A549 cells with or without NAT10 KD. We found that NAT10 KD leads to significant upregulation of IFN-β and ISG transcripts as well as other cytokines/chemokines (Fig. 3B and C; Table S1). KEGG and GO analyses revealed that NAT10 depletion upregulates the expression of genes enriched in the type I IFN-related pathways, including cytokine-cytokine receptor interaction, JAK-STAT signaling, toll-like receptor signaling, and RIG-I-like receptor signaling (Fig. 3D and E). This enrichment of type I IFN-related pathways depended on the activation of RNA sensing pathways, as it did not occur in NAT10 KD cells without poly(I:C) stimulation (Fig. S3C and D). Therefore, our transcriptomic analysis suggested that NAT10 suppresses type I IFN production and ISGs triggered by viral RNA sensing pathways.

From the RNA-seq analysis, we noticed that IFN-β is a top hit that is highly upregulated due to NAT10 depletion in poly(I:C)-stimulated A549 cells. It is known that IFN-β is one of the primary cytokines that drive the induction of downstream ISGs through activation of the JAK-STAT pathway (26–28). Thus, we postulated that IFN-β is a major gene target of NAT10 to drive the suppression of antiviral immune responses. We thus confirmed that NAT10 siRNA KD indeed increases IFN-β production in A549 cells treated with poly(I:C), as demonstrated by both protein immunoblotting (Fig. 3F) and RT-qPCR (Fig. S3E). We also confirmed that, beyond A549 cells, NAT10 depletion increases the IFN-β transcript in poly(I:C)-treated HeLa-MAGI cells by RT-qPCR (Fig. S3F). We further showed that NAT10 KD leads to the increase of secreted IFN-β protein by all tested cells stimulated with poly(I:C), including A549 (Fig. S3G), HeLa-MAGI (Fig. S3H), AALE (Fig. 3G), and HSAEC (Fig. 3H), measured by enzyme-linked immunosorbent assay (ELISA). In addition, we determined the effect of NAT10 overexpression on IFN-β production, showing that NAT10 WT decreases poly(I:C)-induced IFN-β production while G641E mutant exerts much weaker effects (Fig. S3I). We thus concluded that NAT10 universally suppresses viral RNA sensing-induced IFN-β production in all tested cell types, and that this likely explains observed decreases in ISGs.

### NAT10 plays a role in silencing IFN-β transactivation via interfering with IRF3

**Fig 4:**
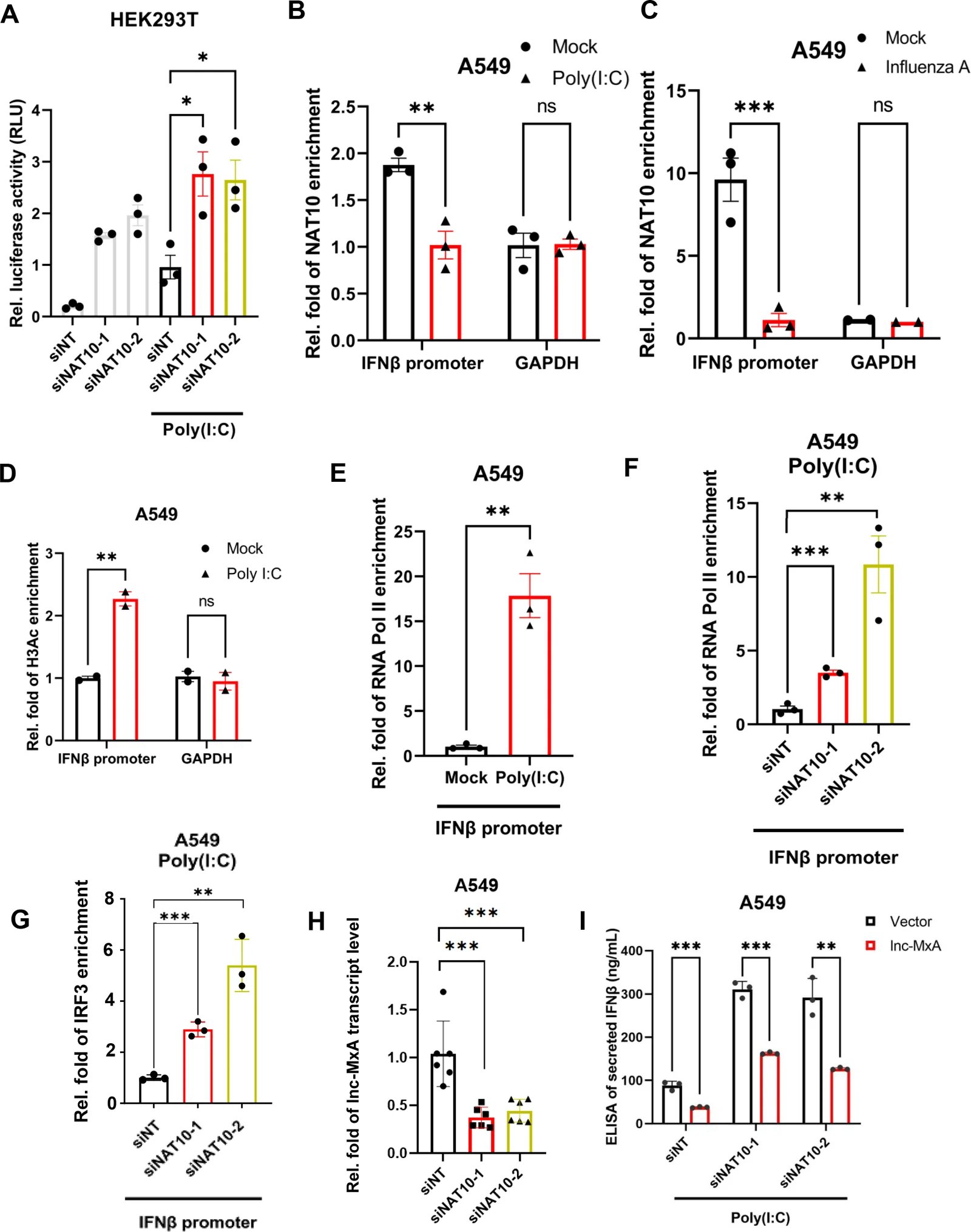
NAT10 contributed to silencing of IFN-β transactivation via interfering with IRF3 activity. (**A**) HEK293T cells were transfected with indicated siRNAs, followed by transfection of IFN-β-luciferase firefly reporter vector, renilla luciferase vector, and RIG-I vector. Cells were further treated with poly(I:C) (1 μg/mL). Luminescence of firefly and renilla luciferases were quantified, and relative light units (RLU) were calculated. (**B and C**) A549 cells were either treated with poly(I:C) (**B**) or challenged with influenza (**C**). Cell samples were prepared and processed for ChIP-PCR to determine NAT10 association at the promoter region of IFN-β. (**D and E**) Cell samples in panel **B** were processed for ChIP-PCR to determine the H3Ac (**D**) and RNA Pol-II (**E**) association at the promoter region of IFN-β. (**F and G**) A549 cells were transfected with indicated siRNAs and treated with poly(I:C) (1 μg/mL), which were processed for ChIP-PCR to determine the RNA Pol-II (**F**) or IRF3 (**G**) association at the promoter region of IFN-β. (**H**) A549 cells were transfected with indicated siRNAs, followed by RNA extractions and RT-qPCR assays to quantify the RNA level of lnc-MxA. (**I**) A549 cells were transfected with the indicated siRNAs, followed by the lentiviral transduction for lnc-MxA overexpression as well as poly(I:C) treatment 24 h post-transduction. The supernatants were collected 24 h after poly(I:C) treatment, and IFN-β was quantified by ELISA. The results were presented as the Mean ± SEM of at least three biological replicates; ns: not significant, *P < 0.05, **P < 0.01, ***P < 0.001, ANOVA and Bonferroni multiple comparison test for (**A**), two-way ANOVA for panels **B–E and I**, or two-tailed Student's *t*-test for panels **F–H**.

Since our data indicated that NAT10 suppresses IFN-β induction at the transcriptional level, we performed a reporter luciferase assay to determine the impact of NAT10 on the IFN-β promoter activity (29). HEK293T cells were co-transfected with an IFN-β promoter-driving firefly luciferase vector, a vector expressing renilla luciferase for normalization, along with a plasmid expressing the poly(I:C) sensor RIG-I. These cells were also transfected with siRNAs targeting NAT10 and stimulated with poly(I:C). Luciferase quantification indicated that NAT10 KD increases the relative IFN-β promoter activity (Fig. 4A). We then sought to identify the underlying mechanisms by which NAT10 decreases IFN-β promoter activity. We found that NAT10 physically associates with the IFN-β promoter in A549 cells, which is supported by the previously published ChIP-seq data of NAT10 (30), and such interaction was significantly reduced upon poly(I:C) stimulation (Fig. 4B) or IAV infection (Fig. 4C).

Notably, NAT10 was initially identified as a histone acetyltransferase that catalyzes histone modification, including the acetylation of histone H3 (H3Ac), which generally associates with the transcriptional activation of genes (31, 32). Thus, we explored the role of NAT10 in histone modification near the IFN-β promoter and found that NAT10 KD has no obvious effect on the local H3Ac level at the IFN-β promoter nor its chromatin accessibility (Fig. S4A and B). However, despite NAT10 dissociation from the IFN-β promoter (Fig. 4B), H3 acetylation at the IFN-β promoter increased in poly(I:C)-treated A549 cells (Fig. 4D), indicating that NAT10 is not likely to be the dominant regulator of histone acetylation at the IFN-β promoter. Interestingly, NAT10 depletion enhanced the recruitment of RNA polymerase II (Pol II) at the IFN-β promoter induced by poly(I:C) (Fig. 4E and F).

It is known that IRF3 is a key transcription factor responsible for IFN-β induction. IRF3 can be activated due to its phosphorylation by the upstream kinase TBK1. Such activation further leads to IRF3 dimerization and nuclear translocation, as well as its binding to the IFN-β promoter to transactivate its expression by further recruiting RNA Pol II. We thus determined the impact of NAT10 on IRF3 activity. Similar to RNA Pol II, NAT10 depletion increased the association of IRF3 with the IFN-β promoter (Fig. 4G). We further confirmed that although overexpression of NAT10 WT significantly decreases the IRF3 association at the IFN-β promoter, the NAT10 acetyltransferase activity-deficient mutant G641E has no obvious effects (Fig. S4C). However, depletion of NAT10 failed to generate any significant effects on the level of either phosphorylated IRF3 or its protein dimerization in A549 cells treated with poly(I:C) or infected with VSV (Fig. S4D and E). In addition, we determined the effect of NAT10 depletion on IFN-β induction in the presence of constitutively active IRF3-5D as well as the association of IRF3-5D at the IFN-β promoter. The results showed that NAT10 depletion not only enhances the IFN-β expression caused by IRF3-5D overexpression but also IRF3-5D association at the IFN-β promoter, indicating that NAT10 likely blocks IFN-β induction at the step post IRF3 activation/phosphorylation (Fig. S4F and G). IRF3 was reported to directly induce gene expression other than IFN-β (33). To determine whether NAT10 specifically affects IRF3 association at the IFN-β promoter, we measured the IRF3 association at the ISG15 promoter, which is reported as an IRF3 target. The results showed that NAT10 depletion only modestly reduces IRF3 binding to the ISG15 promoter (Fig. S4H), indicating that the NAT10-mediated interference with IRF3 chromatin association is context-dependent and likely specific to IFN-β. Next, we sought to determine whether NAT10 affects IFN-β promoter activities through regulation of other transcription factors beyond IRF3, such as NF-κB, by using the PRDII-Luc reporter that contains the NF-κB responding element of the IFN-β promoter to drive luciferase expression. Results showed that NAT10 depletion increases the luciferase signals induced by the constitutively active RIG-I 2CARD transfected in HEK293T cells (Fig. S4I), indicating that NAT10 could interfere with the IFN-β promoter activities beyond IRF3, which may also involve NF-κB.

Lnc-MxA has been reported to associate with the IFN-β promoter and suppress its transcriptional activation by inhibiting the binding of IRF3 (8). Correlated with these findings, we observed that depletion of NAT10 also significantly reduced the level of IRF3-antagonizing lncRNA lnc-MxA in A549 cells both with and without poly(I:C) (Fig. 4H; Fig. S4J). To further understand how NAT10 regulates lnc-MxA, we measured the RNA stability of lnc-MxA in HEK293T cells with NAT10 depletion by siRNA. As shown, the RNA stability of lnc-MxA decreased significantly due to NAT10 knockdown (Fig. S4K). To determine whether NAT10-mediated IFN-β suppression is indeed through lncMxA, we overexpressed the lncMxA RNA in A549 cells with NAT10 depletion by siRNA, followed by the measurement of secreted IFN-β in supernatants using ELISA. As shown (Fig. 4I; Fig. S4L), although NAT10 knockdown increased the poly(I:C)-induced IFN-β production, the lncMxA overexpression significantly counteracted such effects, suggesting that lncMxA plays a critical role in NAT10-mediated IFN-β suppression.

Our findings support a model wherein NAT10 suppresses IFN-β expression by silencing its promoter activity through its interference with IRF3 chromatin association at the IFN-β promoter as well as IRF3-mediated RNA Pol II recruitment.

### NAT10 inhibition induces IFN-β expression while inhibiting viral infection

**Fig 5:**
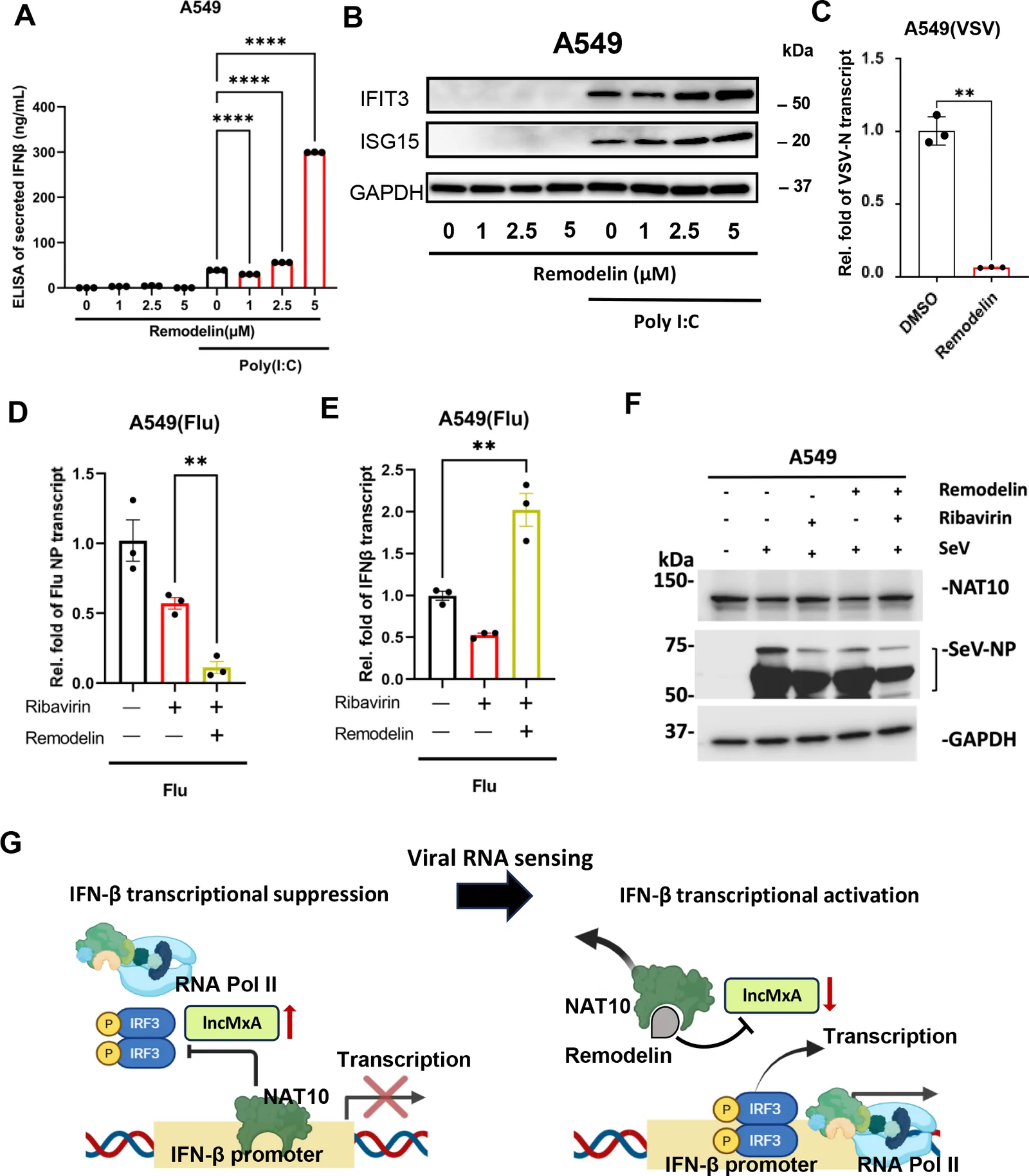
NAT10 inhibitor Remodelin induces IFN-β expression while restricting viral infection. (**A**) A549 cells were treated with Remodelin at a series of doses (0, 1, 2.5, and 5 μM), followed by poly(I:C) stimulation. Cell supernatants were collected for quantification of secreted IFN-β protein by ELISA. (**B**) Cell samples in panel **A** were subjected to immunoblotting to quantify ISG proteins. GAPDH was used as a loading control. (**C**) Expression of VSV-N in A549 cells infected with VSV and treated with Remodelin (5 μM) was measured by RT-qPCR. (**D and E**) A549 cells were treated with ribavirin (10 μM) alone or in combination with Remodelin (5 μM), followed by challenge with influenza viruses. Expression of flu NP (**D**) or IFN-β (**E**) was measured by RT-qPCR. (**F**) A549 cells were treated with ribavirin (10 μM) alone or in combination with Remodelin (5 μM), and infected with Sendai virus (SeV). NAT10 or SeV-NP protein was measured by immunoblotting using their specific antibodies. GAPDH was used as a loading control. (**G**) A working model that NAT10 suppresses viral RNA sensing induced IFN-β transcriptional activation through interfering with IRF3 activity at the IFN-β promoter by modulating IRF3-antagonizing lncRNA lnc-MxA, which can be disrupted by the NAT10 inhibitor Remodelin to argue the antiviral immune responses. The results were presented as the Mean ± SEM; **P < 0.01, ****P < 0.0001, ANOVA and Bonferroni multiple comparison test for panel **A** or two-tailed Student *t*-test for panels **C–E**.

Given that we identified that NAT10 supports viral infection through suppression of IFN-β expression, NAT10 could be considered a new host target for the development of antiviral immunotherapies. It has been reported that Remodelin hydrobromide, 4-[2-(2-cyclopentylidenehydrazinyl)−4-thiazolyl]benzonitrile, acts as a small-molecule inhibitor of NAT10 by targeting its acetyl group binding site (34, 35). We first determined whether Remodelin treatment affects lnc-MxA since it inhibits NAT10. We treated A549 cells with Remodelin, followed by RT-qPCR to quantify lnc-MxA. Results showed that, as a NAT10 inhibitor, Remodelin indeed reduces lnc-MxA RNA levels similar to NAT10 depletion (Fig. S5A). We thus evaluated the antiviral potential of Remodelin in cell cultures. A549 cells were stimulated with poly(I:C) and treated with Remodelin, which caused a dose-responsive increase in secreted IFN-β measured by ELISA (Fig. 5A) and an increase in the induction of ISGs measured by protein immunoblotting (Fig. 5B). We next determined whether the use of Remodelin can inhibit the infection of RNA viruses. Our results showed that Remodelin potently blocked VSV infection in A549 cells through measurement of VSV-N expression by RT-qPCR (Fig. 5C).

We also tested the antiviral potency of Remodelin on other RNA viruses, including IAV and Sendai virus (SeV). However, Remodelin treatment alone had no inhibitory effect on IAV infection or the induction of IFN-β in A549 cells (Fig. S5B and C). We further tested the anti-IAV potential of Remodelin in another scenario by combining it with other antiviral reagents, such as Ribavirin. Ribavirin is an FDA-approved drug that is used together with pegylated IFNs in clinics as the standard treatment for chronic hepatitis C virus (HCV) infection, which also potently inhibits IAV infection (36, 37). We showed that Ribavirin in combination with Remodelin renders a stronger antiviral potency to block IAV NP expression in A549 cells compared to Ribavirin alone (Fig. 5D). Interestingly, inhibition of viral replication by Ribavirin also restored the ability of Remodelin to increase IFN-β induction (Fig. 5E). Similarly, the combination of Remodelin with Ribavirin decreased SeV NP levels while neither of them alone had such effects (Fig. 5F). Thus, by using Remodelin as a chemical probe of NAT10, our studies illustrated that NAT10 inhibition could potentially suppress viral infection by efficiently inducing the activation of antiviral type I IFN signaling.

## DISCUSSION

Recently, there is mounting evidence, including our own data (Fig. 1–3), suggesting that NAT10 plays a critical role in regulating virus-host interactions as well as antiviral innate immune sensing and responses (11–15). Overall, our studies identified that NAT10 suppresses transcriptional activation of IFN-β that is induced by viral RNA sensing through interfering with IRF3 activity by modulating IRF3-antagonizing lncRNA (Lnc-MxA), thus favoring infection of RNA viruses (Fig. 5G). NAT10 was initially identified as an enzyme that acetylates histones (H3 and H4) and non-histone proteins, but it has also been recently illustrated to catalyze the formation of ac4C on mRNAs (24, 32, 38). Histone acetylation at multiple lysine sites is generally associated with transcriptional activation (31, 39, 40), indicating that NAT10’s function to suppress IFN-β might be independent of its histone acetyltransferase activity. NAT10 depletion appeared to have no effect on reducing local histone H3 acetylation nor chromatin accessibility at the IFN-β promoter (Fig. S4A and B). However, H3 acetylation at the IFN-β promoter still increased upon poly(I:C) treatment (Fig. 4D), consistent with previously reported results (10, 41).

Our own results showed that NAT10 associates with the IFN-β promoter and that NAT10 depletion enhances the recruitment of RNA Pol II to the IFN-β promoter, reminiscent of the similar effect of poly(I:C) stimulation (Fig. 4B–F). Thus, we explored other potential mechanisms that might involve NAT10-mediated type I IFN suppression. Beyond histones and chromatin remodeling factors, the transcription factor IRF3 can mediate RNA Pol II association and determine the outcome of IFN-β expression. Once IRF3 is phosphorylated and activated, it translocates to the nuclei and binds to the IFN-β promoter, which is critical to type I IFN immune responses. We identified a novel mechanism by which NAT10 reduces IRF3 association with the IFN-β promoter but not IRF3 phosphorylation and dimerization (Fig. 4G, Fig. S4D and E). It is plausible that NAT10 acts through its RNA acetyltransferase functions by targeting RNA species that antagonize IRF3-mediated IFN-β induction. RNA ac4C acetylation by NAT10 may thus play a role in regulating the type I IFN signaling pathway. The evidence has emerged that RNA modifications, including acetylation, contribute to the balance of immune/inflammatory responses as well as the progression of autoimmune and infectious diseases (42). It is known that the noncoding RNAs (ncRNAs), including long ncRNAs (lncRNAs), undergo epi-transcriptomic modifications, including RNA acetylation (43–45). It will be interesting to characterize NAT10-mediated ac4C modifications of lncRNAs and whether this influences the ability to antagonize IRF3-mediated IFN-β induction. At present, we have demonstrated that NAT10 reduces IFN-β production induced by poly(I:C), while acetyltransferase activity-deficient mutant G641E has much weaker effects (Fig. S3I). NAT10 also increases the RNA level via promotion of RNA stability of the reported IRF3-antagonizing lncRNA lnc-MxA (Fig. 4H**;** Fig. S4J). A recent report showed that NAT10 enhances gene expression by regulating p300/CBP complex (46). While we demonstrated that NAT10 is required for RNA stability of lnc-MxA (Fig. S4K), it is possible that NAT10 may additionally contribute to transcriptional activation of lnc-MxA through regulation of p300/CBP, which needs more investigations in the future.

Beyond the mechanisms identified from our studies that NAT10 inhibits the IRF3-mediated IFN-β transactivation, there could be alternative mechanisms that potentially explain functions of NAT10 in the type I IFN signaling pathway (Fig. 3). For instance, it has been reported that NAT10 activates the transcription repressor MORC2 to downregulate CDK1 and cyclin B1 gene expression during DNA damage (47). It has also been shown that p53 is acetylated by NAT10, which impacts p53 transcriptional activities (48–50). There is evidence that p53 influences regulates IFN-β expression (51). Beyond NAT10’s direct association with the IFN-β promoter to regulate its expression (Fig. 4), NAT10 might also interfere with the upstream factors/players in type I IFN induction, thus adding another layer of regulatory mechanism. For example, it has been reported that NAT10 dysregulates STING activation and thus inhibits type I IFN signaling in a sepsis mouse model (16). Therefore, NAT10 likely suppresses IFN-β induction through multiple mechanisms either directly or indirectly, which requires additional detailed investigations. Our result showed that NAT10 also targets the NF-κB response element in IFN-β promoter (Fig. S4I), which might further contribute to IFN-β inhibition by NAT10.

Since we showed that NAT10 depletion enhances type I IFN induction while restricting infection of multiple RNA viruses (Fig. 1 and 2), we speculated that its inhibition would generate the antiviral benefits. Remodelin is a reported small-molecule inhibitor that targets the NAT10 acetyl-binding site (35). We demonstrated that NAT10 inhibition by Remodelin not only causes IFN-β induction (Fig. 5A, B, and E) but also blocks viral infection (Fig. 5C, D, and F), indicating that its acetyltransferase function could be critical to the immune response and/or viral infection. Although Remodelin has been questioned as a NAT10 inhibitor by a previous study (52), there are also several studies that confirmed its function to inhibit NAT10 (22, 53–57). Our result supported that NAT10 inhibition by Remodelin decreases lnc-MxA RNA level while increasing IFN-β production similar to NAT10 knockdown by siRNA (Fig. 4H and 5A, B, and E; Fig. S5A). However, Remodelin has been reported to inhibit both NAT10 RNA and protein acetyltransferase activities (34, 47, 58, 59). Although still unavailable, a more specific inhibitor that would only target NAT10 RNA acetyltransferase activity is preferred to confirm our findings. We noticed that Remodelin is sufficient to inhibit infection of certain RNA viruses (VSV) on its own while it exerts the antiviral effect on other viruses (IAV and SeV) only in combination with an antiviral. It is possible that only inhibition of NAT10 without its protein depletion would not be sufficient to result in an antiviral effect for certain viruses. It has been reported that the viral proteins of IAV, such as PB1, interact with NAT10 (13, 60), which could manipulate its functions (61, 62). Ribavirin has been implicated to cause the mutagenesis of IAV’s viral genome (63), which may synergize with Remodelin, which inhibits NAT10 enzymatic activities. However, such mechanisms still need further studies.

Overall, our study revealed the novel functions of NAT10 in dampening the viral RNA sensing-induced antiviral innate immune response, thus favoring replication of RNA viruses. We further identified a new mechanism for NAT10 to suppress IFN-β transactivation through interfering with IRF3 activity by modulating IRF3-antagonizing lncRNAs. Finally, as a proof of principle, we showcased that the inhibition of NAT10 by using a small-molecule compound Remodelin results in a potent antiviral effect by inducing type I IFN signaling. These findings warrant further investigations of NAT10 as a promising host target for augmenting antiviral innate immune responses and the further developments of its small-molecule inhibitors for antiviral immunotherapies.

## MATERIALS AND METHODS

### Cell culture

A549 (Cat. #CCL-185, ATCC) is cultured in Ham’s F12K (Cat. #21127022, Gibco). HeLa-MAGI (NIH AIDS Reagent Repository) and HEK293T (Cat. #CRL-3216, ATCC) are cultured in Dulbecco’s modified Eagle’s medium (DMEM; Cat. #D5796, Sigma). The cell culture media above contained 10% fetal bovine serum (FBS; Cat. #10437028, Thermo Fisher), penicillin (100 U/mL)/streptomycin (100 μg/mL) (Cat. #MT30002CI, Corning). HSAEC (Cat. #PCS-301-010, ATCC) are cultured in Airway Epithelial Cell Basal Medium (Cat. #PCS-300-030, ATCC) supplemented with Bronchial Epithelial Growth Kit (Cat. #PCS-300-040, ATCC). Immortalized tracheobronchial epithelial (AALE) cells are cultured in SAGM medium containing supplement kit (Cat. #CC-3118, Lonza).

### Compounds and antibodies

Immuno-stimulant Poly(I:C) (Cat. #528906, Merck) was resuspended in water. Remodelin (Cat. #HY16706A, MedChemExpress) was resuspended in DMSO. Ribavirin (Cat. #R0077, Tokyo Chemical Industry) was resuspended in water. The following antibodies were used for protein immunoblotting: anti-NAT10 (Cat. #13365-1-AP, Proteintech), anti-IFI16 (Cat. #sc-8023, Santa Cruz Biotechnology), anti-IFIT3 (Cat. #sc-393512, Santa Cruz Biotechnology), anti-ISG15 (Cat. #sc-166755, Santa Cruz Biotechnology), anti-MX1 (Cat. #sc-271399, Santa Cruz Biotechnology), anti-IFITM1 (Cat. #60074-1-lg, Proteintech), anti-IRF3 (Cat. #4302S, Cell Signaling Technology [CST]), anti-phospho-IRF3(Ser386) (Cat. #37829S, Cell Signaling Technology), anti-RNA Polymerase II (Cat. #39097, Active Motif), anti-GAPDH (Cat. #sc-47724, Santa Cruz Biotechnology), anti-H3 (Cat. #61800, Active Motif), anti-H3ac (Cat. #61937, Active Motif), anti-N4-acetylcytidine antibody (Cat. #EPRNCI-184-128, ABCAM), anti-NP (Cat. #GTX125989, GeneTex), goat HRP-conjugated anti-mouse IgG antibody (Cat. #7076S, CST), and goat HRP-conjugated anti-rabbit IgG antibody (Cat. #7074S, CST).

### Virus infection

Sendai virus (SeV) and influenza A virus (A/WSN/1933 [H1N1] strain) were kindly provided by Dr. Toru Takimoto at the University of Rochester School of Medicine and Dentistry. Vesicular stomatitis virus (VSV) was purchased (Cat. #VR-3340, ATCC). When cells reached >80% confluence, influenza A viral stock (MOI = 1) was added to cell medium and incubated with cells for 36 or 48 h, respectively. SeV and VSV viral stock (MOI = 1) was incubated with cells for 24 h.

### Cell transfection

Poly(I:C) was transfected with the Fugene-6 transfection reagent (Cat. #E2691, Promega). Cells were incubated with Poly(I:C) for 24 h. For gene knockdown, siRNA (10 nM) was reversely transfected into cells using Lipofectamine RNAiMAX transfection reagent (Cat. #13778030, Invitrogen) for 72 h. The following siRNAs were used: siNAT10-1 (Cat. #S30491, Life Technologies) and siNAT10-2 (Cat. #S30492, Life Technologies). For the negative control, we used the Silencer Negative Control No. 4 siNT (Cat. #AM4641, Invitrogen).

### Luciferase assay

HEK293T cells were transfected with siRNAs. At 72 h post-transfection, cells were transfected with plasmids including pGL3-IFN-β-luciferase reporter vector (Cat. #102597, Addgene) or PRDII-Luc (kindly provided by Dr. Jacob S. Yount, Ohio State University) and pRL-TK renilla luciferase vector (Cat. #E2241, Promega) along with pTRIP-SFFV-mtagBFP-2A RIGI WT (Cat. #167289, Addgene) or pEBG-RIG-I 2CARD-GST (kindly provided by Dr. Michaela U. Gack, Cleveland Clinic) using the TurboFect transfection reagent (Thermo Scientific) for 24 h. For pGL3-IFN-β-luciferase transfected cells, they were treated with poly(I:C) for another 24 h. The Dual-Glo luciferase system (Cat. #E2920, Promega) was used for quantification of firefly and renilla luminescence. Luminescence was measured by the Cytation 5 multimode reader (BioTek). Relative luciferase unit (RLU) was calculated by normalizing firefly by renilla signal.

### Protein immunoblotting

Cells were lysed in the RIPA buffer (Cat. #20-188, Millipore) containing protease inhibitor cocktail (Cat. #A32965, Thermo Scientific) on ice. With brief sonication, the total protein amount in cell lysate was measured using BCA assay kit (Cat. #23225, Thermo Scientific) for loading normalization. Lysates were mixed with the SDS loading buffer containing 5% β-mercaptoethanol (Cat. #60-24-2, Acros Organics) and boiled. Protein samples were loaded on the Novex WedgeWell 4–20% SDS-PAGE Tris-Glycine gel (Invitrogen) or the Mini-Protean TGX gel (Bio-Rad), and transferred to the iBlot 2 Transfer Stacks PVDF (Invitrogen) or the RTA transfer kit PVDF (Bio-Rad) using the iBlot 2 Dry Blotting System (Cat. #IB21001, Thermo Scientific) or the Trans-blot Turbo (Cat. #1704150, Biorad). Membranes were blocked with 5% BSA in PBS for 1 h and incubated with the primary antibodies at 4°C overnight, followed by incubation with HRP-conjugated secondary antibodies for 1 h. The membranes were washed three times with PBST (PBS with 0.5% Tween 20). The membranes were developed using the Clarity Max ECL substrate (Cat. #1705062, Bio-Rad).

### Enzyme-linked immunosorbent assay (ELISA)

Human IFN-β DuoSet ELISA (Cat. #DY814-05, R&D Systems) was used according to the manufacturer’s protocols. Briefly, supernatants were collected from cultured cells. ELISA plates were coated with capture antibody overnight, followed by incubation with blocking buffer for 1 h, supernatant for 2 h, detection antibody for 2 h, streptavidin-HRP for 20 min, and stopping solution for 10 min. Between each incubation, ELISA plates were washed four times with the washing buffer. Absorbance at 450 nm was quantified using the Cytation 5 multimode reader. IFN-β concentrations were calculated based on a standard curve.

### RNA stability assays

HEK293T cells were transfected with siNT, siNAT10-1, or siNAT10-2 for 36 h, followed by poly(I:C) (1 μg/mL) treatment for another 24 h. Cells were then treated with actinomycin D (10 μg/mL) in fresh medium. Cell samples were collected at 0, 1, 2, 3, 6, and 10 h after drug treatment and subjected to RNA extraction. The RNA samples were analyzed by qPCR assays. The relative level of lnc-MxA RNA was normalized to 18S rRNA.

### Cell viability assay

Cell viability was measured using ATP-based CellTiter-Glo (Cat. #G7571, Promega) by following the manufacturer’s instructions. Briefly, Cell-Titer-Glo was added to each cell in media at a 1:1 ratio in the opaque 96-well plate. The plate was incubated for 10 min on a rocking shaker protected from the light. The luminescent signal was analyzed by the Cytation 5 reader.

### Chromatin immunoprecipitation (ChIP) assay

Cells were cross-linked with 0.5% paraformaldehyde, and the reaction was quenched using 100 mM glycine. Cell lysis was performed on ice for 10 min in CE buffer (10 mM HEPES-KOH, 60 mM KCl, 1 mM EDTA, 0.5% NP-40, and 1 mM DTT, pH 7.9) supplemented with a protease inhibitor cocktail. Nuclei were pelleted in SDS lysis buffer (1% SDS, 10 mM EDTA, and 50 mM Tris-HCl, pH 8.1) containing protease inhibitors by centrifugation at 700 × *g* for 10 min at 4°C. Nuclear lysates in SDS buffer were subjected to sonication for 2 min to fragment genomic DNAs and diluted with the ChIP dilution buffer (0.01% SDS, 1% Triton X-100, 1.2 mM EDTA, 16.7 mM Tris-HCl, and 150 mM NaCl, pH 8.1) containing protease inhibitors. The lysates were incubated overnight at 4°C with specific antibodies or control rabbit IgG (Cat. #sc-2025, Santa Cruz Biotechnology). Protein A/G beads (Cat. #88803, Pierce) were pre-blocked with 0.5 mg/mL BSA and 0.125 mg/mL herring sperm DNA (Cat. #15634-017, Invitrogen) at room temperature for 1 h and then added to the mixture for incubation for another 2 h at 4°C. The beads were washed sequentially with the following buffers: low salt wash buffer (0.1% SDS, 1% Triton X-100, 2 mM EDTA, 20 mM Tris-HCl, and 150 mM NaCl, pH 8.1); high-salt wash buffer (0.1% SDS, 1% Triton X-100, 2 mM EDTA, 20 mM Tris-HCl, and 500 mM NaCl, pH 8.1); LiCl buffer (0.25 M LiCl, 1% NP-40, 1% Na-deoxycholate, 1 mM EDTA, and 10 mM Tris-HCl, pH 8.1); and TE buffer (10 mM Tris-HCl and 0.1 mM EDTA, pH 8.1). Protein samples were eluted using the fresh elution buffer (1% SDS, 0.1 M NaHCO_3_) at room temperature. The eluates were incubated at 65°C overnight with 0.2 M NaCl to dissociate protein-DNA complexes, followed by treatment with proteinase K (Cat. #EO0491, Thermo Fisher Scientific) at 50°C for 2 h for protein digestion. DNAs were extracted using UltraPure Phenol:Chloroform:Isoamyl Alcohol (Cat. #15593031, Thermo Fisher Scientific) by following the manufacturer’s instructions, and the resulting DNA pellets were resuspended in water for subsequent qPCR analysis.

### Quantitative PCR (qPCR)

Total RNAs were extracted from cells using the NucleoSpin RNA extraction kit (Cat. #740955.250, MACHEREY-NAGEL). Subsequently, 500 ng of RNAs was reverse transcribed into cDNAs using the iScript cDNA synthesis kit (Cat. #1708890, Bio-Rad). Real-time qPCR was carried out using the iTaq Universal SYBR Green Supermix (Cat. #1727125, Bio-Rad) on a Bio-Rad CFX Connect qPCR system. The thermal cycling conditions were as follows: an initial denaturation step at 95°C for 10 mins, followed by 50 cycles of 95°C for 15 s and 60°C for 1 min. The relative cDNA level of the gene was normalized to GAPDH using the 2^−ΔΔCt^ method: 2^(ΔCT of targeted gene − ΔCT of GAPDH)^. Values were normalized to the control using the same method: 2^(ΔCT of targeted gene − ΔCT of IgG pull-down)^. The qPCR primers were listed (Table S2).

### RNA sequencing (RNA-seq) analysis

Total RNAs were extracted from cells using the NucleoSpin RNA extraction kit. RNA samples were submitted to GENEWIZ (South Plainfield, NJ, USA) for quality control and further procedures. Briefly, RNA samples were processed with rRNA removal via poly-A selection, followed by library preparation. Sequencing was performed on an Illumina HiSeq platform using a 2 × 150 bp paired-end configuration, achieving more than 20 million reads per cell sample. Differential gene expression analysis was conducted using the DESeq2 package. Heatmaps and pathway analysis were generated with the R package pheatmap and the clusterProfiler package.

### Immunofluorescence assay (IFA)

Cells were fixed with 4% paraformaldehyde for 10 min at room temperature and permeabilized with 0.2% Triton X-100 for 15 min. Blocking was performed with D-PBS containing 5% FBS for 1 h. Cells were incubated overnight at 4°C with the primary antibodies in D-PBS containing 2.5% FBS, followed by incubation for 1 h at RT with Alexa Fluor 488-conjugated (Cat. #A11008, Invitrogen) or Alexa Fluor 568-conjugated (Cat. #A11004, Invitrogen) secondary antibodies. Nuclei were stained with Hoechst 33342 (Thermo Scientific) for 10 min at room temperature. Imaging was carried out using a confocal microscope, and the percentage of virus-infected cells was quantified using ImageJ.

### Statistics

Statistical analysis was performed using GraphPad PRISM. Data were presented as Mean ± standard error of the mean (SEM) of at least three biological replicates. *P < 0.05, **P < 0.01, ***P < 0.001, or ****P < 0.001 indicated a significant difference analyzed by unpaired two-tailed *t*-test, one-way, or two-way ANOVA, and Bonferroni multiple comparison test.

## Data Availability

All data needed to evaluate the conclusions in the paper are present in the paper and/or the supplemental material. Additional data and materials related to this paper may be requested from the authors.

## SUPPLEMENTAL MATERIAL

Supplemental Figures mbio.01452-26-s0001.docx Fig. S1 to S5.

Supplemental Tables mbio.01452-26-s0002.xlsx Table S1 and S2.

ASM does not own the copyrights to Supplemental Material that may be linked to, or accessed through, an article. The authors have granted ASM a non-exclusive, world-wide license to publish the Supplemental Material files. Please contact the corresponding author directly for reuse.

## References

[1] Rehwinkel J, Gack MU. 2020. RIG-I-like receptors: their regulation and roles in RNA sensing. Nat Rev Immunol 20:537–551. doi:10.1038/s41577-020-0288-3 32203325 PMC7094958

[2] AL Hamrashdi M, Brady G. 2022. Regulation of IRF3 activation in human antiviral signaling pathways. Biochem Pharmacol 200:115026. doi:10.1016/j.bcp.2022.115026 35367198

[3] Hiscott J. 2007. Triggering the innate antiviral response through IRF-3 activation. J Biol Chem 282:15325–15329. doi:10.1074/jbc.R700002200 17395583

[4] Lukhele S, Boukhaled GM, Brooks DG. 2019. Type I interferon signaling, regulation and gene stimulation in chronic virus infection. Semin Immunol 43:101277. doi:10.1016/j.smim.2019.05.001 31155227 PMC8029807

[5] Wang A, Hu J, Zhang Q, Zhang Y, Wei F. 2026. The antiviral activity of interferon-stimulated genes (ISGs) in influenza A virus infection. Virology 614:110728. doi:10.1016/j.virol.2025.110728 41175493

[6] Schwanke H, Stempel M, Brinkmann MM. 2020. Of keeping and tipping the balance: host regulation and viral modulation of IRF3-dependent IFNB1 expression. Viruses 12:733. doi:10.3390/v12070733 32645843 PMC7411613

[7] Yu Z, Wang L, Zhao J, Song H, Zhao C, Zhao W, Jia M. 2022. TOB1 attenuates IRF3-directed antiviral responses by recruiting HDAC8 to specifically suppress IFN-β expression. Commun Biol 5:943. doi:10.1038/s42003-022-03911-x 36085336 PMC9463440

[8] Li X, Guo G, Lu M, Chai W, Li Y, Tong X, Li J, Jia X, Liu W, Qi D, Ye X. 2019. Long noncoding RNA Lnc-MxA inhibits beta interferon transcription by forming RNA-DNA triplexes at its promoter. J Virol 93:e00786-19. doi:10.1128/JVI.00786-19 31434735 PMC6803265

[9] Freaney JE, Kim R, Mandhana R, Horvath CM. 2013. Extensive cooperation of immune master regulators IRF3 and NFκB in RNA Pol II recruitment and pause release in human innate antiviral transcription. Cell Rep 4:959–973. doi:10.1016/j.celrep.2013.07.043 23994473 PMC3792498

[10] Agalioti T, Lomvardas S, Parekh B, Yie J, Maniatis T, Thanos D. 2000. Ordered recruitment of chromatin modifying and general transcription factors to the IFN-beta promoter. Cell 103:667–678. doi:10.1016/s0092-8674(00)00169-0 11106736

[11] Tsai K, Jaguva Vasudevan AA, Martinez Campos C, Emery A, Swanstrom R, Cullen BR. 2020. Acetylation of cytidine residues boosts HIV-1 gene expression by increasing viral RNA stability. Cell Host Microbe 28:306–312. doi:10.1016/j.chom.2020.05.011 32533923 PMC7429276

[12] Hao H, Liu W, Miao Y, Ma L, Yu B, Liu L, Yang C, Zhang K, Chen Z, Yang J, Zheng Z, Zhang B, Deng F, Gong P, Yuan J, Hu Z, Guan W. 2022. N4-acetylcytidine regulates the replication and pathogenicity of enterovirus 71. Nucleic Acids Res 50:9339–9354. doi:10.1093/nar/gkac675 35971620 PMC9458434

[13] Furuse Y. 2021. RNA modifications in genomic RNA of Influenza A virus and the relationship between rna modifications and viral infection. Int J Mol Sci 22:9127. doi:10.3390/ijms22179127 34502037 PMC8431438

[14] Yan Q, Zhou J, Wang Z, Ding X, Ma X, Li W, Jia X, Gao SJ, Lu C. 2023. NAT10-dependent N4‐acetylcytidine modification mediates PAN RNA stability, KSHV reactivation, and IFI16-related inflammasome activation. Nat Commun 14. doi:10.1038/s41467-023-42135-3 PMC10564894 37816771

[15] Dang Y, Li J, Li Y, Wang Y, Zhao Y, Zhao N, Li W, Zhang H, Ye C, Ma H, Zhang L, Liu H, Dong Y, Yao M, Lei Y, Xu Z, Zhang F, Ye W. 2024. N-acetyltransferase 10 regulates alphavirus replication via N4-acetylcytidine (ac4C) modiﬁcation of the lymphocyte antigen six family member E (LY6E) mRNA. J Virol 98:e01350-23. doi:10.1128/jvi.01350-23 38169284 PMC10805074

[16] Zhang H, Chen Z, Zhou J, Gu J, Wu H, Jiang Y, Gao S, Liao Y, Shen R, Miao C, Chen W. 2022. NAT10 regulates neutrophil pyroptosis in sepsis via acetylating ULK1 RNA and activating STING pathway. Commun Biol 5:916. doi:10.1038/s42003-022-03868-x 36068299 PMC9448771

[17] Guo G, Shi X, Wang H, Ye L, Tong X, Yan K, Ding N, Chen C, Zhang H, Xue X. 2020. Epitranscriptomic N4-acetylcytidine profiling in CD4+ T cells of systemic lupus erythematosus. Front Cell Dev Biol 8:842. doi:10.3389/fcell.2020.00842 32984334 PMC7483482

[18] Chen X, Hao Y, Liu Y, Zhong S, You Y, Ao K, Chong T, Luo X, Yin M, Ye M, He H, Lu A, Chen J, Li X, Zhang J, Guo X. 2023. NAT10/ac4C/FOXP1 promotes malignant progression and facilitates immunosuppression by reprogramming glycolytic metabolism in cervical cancer. Advanced Science 10. doi:10.1002/advs.202302705 PMC10646273 37818745

[19] Gu Z, Zou L, Pan X, Yu Y, Liu Y, Zhang Z, Liu J, Mao S, Zhang J, Guo C, Li W, Geng J, Zhang W, Yao X, Shen B. 2024. The role and mechanism of NAT10-mediated ac4C modification in tumor development and progression. Med Comm 5:e70026. doi:10.1002/mco2.70026 PMC11617596 39640362

[20] Li G, Ma X, Sui S, Chen Y, Li H, Liu L, Zhang X, Zhang L, Hao Y, Yang Z, Yang S, He X, Wang Q, Tao W, Xu S. 2024. NAT10/ac4C/JunB facilitates TNBC malignant progression and immunosuppression by driving glycolysis addiction. J Exp Clin Cancer Res 43:278. doi:10.1186/s13046-024-03200-x 39363363 PMC11451012

[21] Qin G, Bai F, Hu H, Zhang J, Zhan W, Wu Z, Li J, Fu Y, Deng Y. 2024. Targeting the NAT10/NPM1 axis abrogates PD-L1 expression and improves the response to immune checkpoint blockade therapy. Mol Med 30:13. doi:10.1186/s10020-024-00780-4 38243170 PMC10799409

[22] Liu WC, Wei YH, Chen JF, Xing XL, Jia HX, Yang XY, Huang YJ, Liu XM, Xiao K, Guo XD, Can C, Zhang AM, He N, Zhang HL, Ma DX. 2025. Inhibition of tumor-intrinsic NAT10 enhances antitumor immunity by triggering type I interferon response via MYC/CDK2/DNMT1 pathway. Nat Commun 16:5154. doi:10.1038/s41467-025-60293-4 40461504 PMC12134272

[23] Savidis G, McDougall WM, Meraner P, Perreira JM, Portmann JM, Trincucci G, John SP, Aker AM, Renzette N, Robbins DR, Guo Z, Green S, Kowalik TF, Brass AL. 2016. Brass, identification of zika virus and dengue virus dependency factors using functional genomics. Cell Rep 16:232–246. doi:10.1016/j.celrep.2016.06.028 27342126

[24] Arango D, Sturgill D, Alhusaini N, Dillman AA, Sweet TJ, Hanson G, Hosogane M, Sinclair WR, Nanan KK, Mandler MD, Fox SD, Zengeya TT, Andresson T, Meier JL, Coller J, Oberdoerffer S. 2018. Acetylation of cytidine in mRNA promotes translation efficiency. Cell 175:1872–1886. doi:10.1016/j.cell.2018.10.030 30449621 PMC6295233

[25] Debnath TK, Abell NS, Li YR, Devanathan SK, Navedo E, Xhemalçe B. 2025. NAT10 and N(4)-acetylcytidine restrain R-loop levels and related inflammatory responses. Sci Adv 11. doi:10.1126/sciadv.ads6144 PMC11939041 40138394

[26] Ivashkiv LB, Donlin LT. 2014. Regulation of type I interferon responses. Nat Rev Immunol 14:36–49. doi:10.1038/nri3581 24362405 PMC4084561

[27] Wittling MC, Cahalan SR, Levenson EA, Rabin RL. 2020. Shared and unique features of human interferon-beta and interferon-alpha subtypes. Front Immunol 11:605673. doi:10.3389/fimmu.2020.605673 33542718 PMC7850986

[28] Winkler R, Gillis E, Lasman L, Safra M, Geula S, Soyris C, Nachshon A, Tai-Schmiedel J, Friedman N, Le-Trilling VTK, Trilling M, Mandelboim M, Hanna JH, Schwartz S, Stern-Ginossar N. 2019. m6A modification controls the innate immune response to infection by targeting type I interferons. Nat Immunol 20:173–182. doi:10.1038/s41590-018-0275-z 30559377

[29] Linehan MM, Dickey TH, Molinari ES, Fitzgerald ME, Potapova O, Iwasaki A, Pyle AM. 2018. A minimal RNA ligand for potent RIG-I activation in living mice. Sci Adv 4:e1701854. doi:10.1126/sciadv.1701854 29492454 PMC5821489

[30] Zhao W, Liu K. 2021. GEO accession viewer. Available from: https://www.ncbi.nlm.nih.gov/geo/query/acc.cgi?acc=GSE175731

[31] Agalioti T, Chen G, Thanos D. 2002. Deciphering the transcriptional histone acetylation code for a human gene. Cell 111:381–392. doi:10.1016/s0092-8674(02)01077-2 12419248

[32] Lv J, Liu H, Wang Q, Tang Z, Hou L, Zhang B. 2003. Molecular cloning of a novel human gene encoding histone acetyltransferase-like protein involved in transcriptional activation of hTERT. Biochem Biophys Res Commun 311:506–513. doi:10.1016/j.bbrc.2003.09.235 14592445

[33] Grandvaux N, Servant MJ, tenOever B, Sen GC, Balachandran S, Barber GN, Lin R, Hiscott J. 2002. Transcriptional profiling of interferon regulatory factor 3 target genes: direct involvement in the regulation of interferon-stimulated genes. J Virol 76:5532–5539. doi:10.1128/jvi.76.11.5532-5539.2002 11991981 PMC137057

[34] Larrieu D, Britton S, Demir M, Rodriguez R, Jackson SP. 2014. Chemical inhibition of NAT10 corrects defects of laminopathic cells. Science 344:527–532. doi:10.1126/science.1252651 24786082 PMC4246063

[35] Dalhat MH, Altayb HN, Khan MI, Choudhry H. 2021. Structural insights of human N-acetyltransferase 10 and identification of its potential novel inhibitors. Sci Rep 11:6051. doi:10.1038/s41598-021-84908-0 33723305 PMC7960695

[36] Hung IF-N, Lung K-C, Tso EY-K, Liu R, Chung TW-H, Chu M-Y, Ng Y-Y, Lo J, Chan J, Tam AR, et al.. 2020. Triple combination of interferon beta-1b, lopinavir–ritonavir, and ribavirin in the treatment of patients admitted to hospital with COVID-19: an open-label, randomised, phase 2 trial. Lancet 395:1695–1704. doi:10.1016/S0140-6736(20)31042-4 32401715 PMC7211500

[37] Dammacco F, Tucci FA, Lauletta G, Gatti P, De Re V, Conteduca V, Sansonno S, Russi S, Mariggiò MA, Chironna M, Sansonno D. 2010. Pegylated interferon-alpha, ribavirin, and rituximab combined therapy of hepatitis C virus-related mixed cryoglobulinemia: a long-term study. Blood 116:343–353. doi:10.1182/blood-2009-10-245878 20308602

[38] Cao Y, Yao M, Wu Y, Ma N, Liu H, Zhang B. 2020. N-acetyltransferase 10 promotes micronuclei formation to activate the senescence-associated secretory phenotype machinery in colorectal cancer cells. Transl Oncol 13:100783. doi:10.1016/j.tranon.2020.100783 32428852 PMC7232111

[39] Hake SB, Garcia BA, Duncan EM, Kauer M, Dellaire G, Shabanowitz J, Bazett-Jones DP, Allis CD, Hunt DF. 2006. Expression patterns and post-translational modifications associated with mammalian histone H3 variants. J Biol Chem 281:559–568. doi:10.1074/jbc.M509266200 16267050

[40] Brownell JE, Zhou J, Ranalli T, Kobayashi R, Edmondson DG, Roth SY, Allis CD. 1996. Tetrahymena histone acetyltransferase A: a homolog to yeast Gcn5p linking histone acetylation to gene activation. Cell 84:843–851. doi:10.1016/s0092-8674(00)81063-6 8601308

[41] Parekh BS, Maniatis T. 1999. Virus infection leads to localized hyperacetylation of histones H3 and H4 at the IFN-beta promoter. Mol Cell 3:125–129. doi:10.1016/s1097-2765(00)80181-1 10024886

[42] Cui L, Ma R, Cai J, Guo C, Chen Z, Yao L, Wang Y, Fan R, Wang X, Shi Y. 2022. RNA modifications: importance in immune cell biology and related diseases. Signal Transduct Target Ther 7:334. doi:10.1038/s41392-022-01175-9 36138023 PMC9499983

[43] Tsai K, Cullen BR. 2020. Epigenetic and epitranscriptomic regulation of viral replication. Nat Rev Microbiol 18:559–570. doi:10.1038/s41579-020-0382-3 32533130 PMC7291935

[44] Kambara H, Niazi F, Kostadinova L, Moonka DK, Siegel CT, Post AB, Carnero E, Barriocanal M, Fortes P, Anthony DD, Valadkhan S. 2014. Negative regulation of the interferon response by an interferon-induced long non-coding RNA. Nucleic Acids Res 42:10668–10680. doi:10.1093/nar/gku713 25122750 PMC4176326

[45] Carnero E, Barriocanal M, Prior C, Pablo Unfried J, Segura V, Guruceaga E, Enguita M, Smerdou C, Gastaminza P, Fortes P. 2016. Long noncoding RNA EGOT negatively affects the antiviral response and favors HCV replication. EMBO Rep 17:1013–1028. doi:10.15252/embr.201541763 27283940 PMC4931568

[46] Amin R, Ha NH, Qiu T, Holewinski R, Lam KC, Daugherty-Lopès A, Liu H, Tran AD, Lee MP, Andresson T, Goldszmid RS, Hunter KW. 2025. NAT10 promotes cancer metastasis by modulating p300/CBP activity through chromatin-associated tRNA. Mol Cell 85:4526–4544. doi:10.1016/j.molcel.2025.11.010 41344331 PMC12709618

[47] Liu HY, Liu YY, Yang F, Zhang L, Zhang FL, Hu X, Shao ZM, Li DQ. 2020. Acetylation of MORC2 by NAT10 regulates cell-cycle checkpoint control and resistance to DNA-damaging chemotherapy and radiotherapy in breast cancer. Nucleic Acids Res 48:3638–3656. doi:10.1093/nar/gkaa130 32112098 PMC7144926

[48] Liu X, Tan Y, Zhang C, Zhang Y, Zhang L, Ren P, Deng H, Luo J, Ke Y, Du X. 2016. NAT10 regulates p53 activation through acetylating p53 at K120 and ubiquitinating Mdm2. EMBO Rep 17:349–366. doi:10.15252/embr.201540505 26882543 PMC4772976

[49] Li Q, Liu X, Jin K, Lu M, Zhang C, Du X, Xing B. 2017. NAT10 is upregulated in hepatocellular carcinoma and enhances mutant p53 activity. BMC Cancer 17. doi:10.1186/s12885-017-3570-4 PMC5579925 28859621

[50] Qu Z, Pang X, Mei Z, Li Y, Zhang Y, Huang C, Liu K, Yu S, Wang C, Sun Z, et al.. 2024. The positive feedback loop of the NAT10/Mybbp1a/p53 axis promotes cardiomyocyte ferroptosis to exacerbate cardiac I/R injury. Redox Biol 72:103145. doi:10.1016/j.redox.2024.103145 38583415 PMC11002668

[51] Xia W, Jiang P. 2024. P53 promotes antiviral innate immunity by driving hexosamine metabolism. Cell Rep 43:113724. doi:10.1016/j.celrep.2024.113724 38294905

[52] Shrimp JH, Jing Y, Gamage ST, Nelson KM, Han J, Bryson KM, Montgomery DC, Thomas JM, Nance KD, Sharma S, Fox SD, Andressen T, Sinclair WR, Wu H, Allali-Hassani A, Senisterra G, Vedadi M, Lafontaine D, Dahlin JL, Marmorstein R, Walters MA, Meier JL. 2021. Remodelin is a cryptic assay interference chemotype that does not inhibit NAT10-dependent cytidine acetylation. ACS Med Chem Lett 12:887–892. doi:10.1021/acsmedchemlett.0c00193 34141066 PMC8201477

[53] Qin J, Shen Y, Li S, Hu S, Song F, Jin T, Wu J, Xu J. 2026. An ac4C-CDK4 regulatory axis driven by NAT10 sustains proliferative signaling in colorectal cancer. Transl Oncol 69:102807. doi:10.1016/j.tranon.2026.102807 42127733 PMC13196451

[54] Zhang M, Li Y, Wu C, Pan Q, Wang P, Liu HF. 2026. NAT10-mediated ac4C modification of TNFRSF1A promotes acute kidney injury by activating NF-κB pathway. Cell Mol Biol Lett 31. doi:10.1186/s11658-026-00913-z PMC13374305 42121010

[55] Wang Z, Wu Q, Yao Y, Cao M, Ding J, Xiao Y, Huang X, Luo J. 2026. Targeting the RNA acetyltransferase NAT10 attenuates pulmonary fibrosis via the ac4C-mediated regulation of the C/EBPβ-ITGA11 axis. Int Immunopharmacol 181:116793. doi:10.1016/j.intimp.2026.116793 42090905

[56] Gao Q, Qiu L, Xiao M, Li RF, Wang J, Ge XY. 2026. MYB-driven NAT10–IGF2BP3 RNA modification axis sustains MAPK signaling and promotes adenoid cystic carcinoma progression. Int J Biol Macromol 365:152223. doi:10.1016/j.ijbiomac.2026.152223 42067098

[57] Mo X, Meng K, Xu B, Li Z, Lan S, Ren Z, Xiang X, Zou P, Chen Z, Lai Z, Ao X, Liu Z, Shang W, Dai B, Luo L, Xu J, Wang Z, Zhang Z. 2025. Nat10-mediated N4-acetylcytidine modification enhances Nfatc1 translation to exacerbate osteoclastogenesis in postmenopausal osteoporosis. Proc Natl Acad Sci USA 122:e2423991122. doi:10.1073/pnas.2423991122 40193598 PMC12012521

[58] Yang W, Li HY, Wu YF, Mi RJ, Liu WZ, Shen X, Lu YX, Jiang YH, Ma MJ, Shen HY. 2021. ac4C acetylation of RUNX2 catalyzed by NAT10 spurs osteogenesis of BMSCs and prevents ovariectomy-induced bone loss. Mol Ther Nucleic Acids 26:135–147. doi:10.1016/j.omtn.2021.06.022 34513300 PMC8413676

[59] Liu D, Kuang Y, Chen S, Li R, Su F, Zhang S, Qiu Q, Lin S, Shen C, Liu Y, Liang L, Wang J, Xu H, Xiao Y. 2024. NAT10 promotes synovial aggression by increasing the stability and translation of N4-acetylated PTX3 mRNA in rheumatoid arthritis. Ann Rheum Dis 83:1118–1131. doi:10.1136/ard-2023-225343 38724075

[60] Zeng Y, Xu S, Wei Y, Zhang X, Wang Q, Jia Y, Wang W, Han L, Chen Z, Wang Z, Zhang B, Chen H, Lei CQ, Zhu Q. 2021. The PB1 protein of influenza A virus inhibits the innate immune response by targeting MAVS for NBR1-mediated selective autophagic degradation. PLoS Pathog 17:e1009300. doi:10.1371/journal.ppat.1009300 33577621 PMC7880438

[61] Shen Q, Zheng X, McNutt MA, Guang L, Sun Y, Wang J, Gong Y, Hou L, Zhang B. 2009. NAT10, a nucleolar protein, localizes to the midbody and regulates cytokinesis and acetylation of microtubules. Exp Cell Res 315:1653–1667. doi:10.1016/j.yexcr.2009.03.007 19303003

[62] Xiao B, Wu S, Tian Y, Huang W, Chen G, Luo D, Cai Y, Chen M, Zhang Y, Liu C, Zhao J, Li L. 2024. Advances of NAT10 in diseases: insights from dual properties as protein and RNA acetyltransferase. Cell Biol Toxicol 41:17. doi:10.1007/s10565-024-09962-6 39725720 PMC11671434

[63] Crotty S, Maag D, Arnold JJ, Zhong W, Lau JY, Hong Z, Andino R, Cameron CE. 2000. The broad-spectrum antiviral ribonucleoside ribavirin is an RNA virus mutagen. Nat Med 6:1375–1379. doi:10.1038/82191 11100123

