# Supplementary material for: NAT10 is critical to block RNA sensing-induced IFN-β transactivation in viral infection
Source: mBio. 2026 Aug 4;17(9):e01452-26. doi: 10.1128/mbio.01452-26 (PMC13556238; doi:10.1128/mbio.01452-26)
Supplement: Supplementary file 1 [file mbio.01452-26-s0001.docx]

**Figure S1. (A-C)** Viability of HeLa-MAGI (A), A549 (B), and HSAEC (C) cells transfected with indicated siRNAs at day 3 with or without poly(I:C) treatment. The results were presented as the Mean ± S.E.M.; ns, not significant. **P* < 0.05, ANOVA and Bonferroni multiple comparison test. **(D)** A549 cells were transduced with lentivral vectors expressing V5-tagged NAT10 WT or G641E, followed by immunoblotting to quantify protein expression of NAT10 WT or G641E using an anti-V5 antibody. GAPDH was used as a loading control.

**Figure S2. (A)** A549 cells were treated with poly(I:C) at a series of doses (0, 0.05, 0.1, 0.5, 1μg/mL). Expression of NAT10 or IFN-β was measured by RT-qPCR. **(B)** Cell samples in (A) were analyzed by immunoblotting to quantify NAT10 and IFIT3 proteins. GAPDH was used as a loading control. **(C)** Protein expression of NAT10 was visualized by immunofluorescence (Alexa 488) in A549 cells transfected with indicated siRNAs and treated with poly(I:C).

**Figure S3. (A, B)** KEGG (A) and GO (B) analysis of DEGs induced by Poly(I:C) treatment in A549 cells. **(C, D)** Volcanoplot (C) and GO analysis (D) of upregulated DEGs due to NAT10 depletion by its siRNAs in A549 cells without poly(I:C) treatment. **(E, F)** A549 (E) or HeLa-MAGI (F) cells were transfected with indicated siRNAs, followed by the poly(I:C) treatment (1μg/mL). Expression of IFN-β was measured by RT-qPCR. **(G, H)** Cell supernatants from siRNA-transfected A549 (G) or HeLa-MAGI (H) cells were collected to quantify the secreted IFN-β protein by ELISA. **(I)** A549 cells were transduced with V5-tagged NAT10 WT and G641E, followed by poly(I:C) treatment (1μg/mL). Secreted IFN-β was measured by ELISA. The results were presented as the Mean ± S.E.M.; ** *P*< 0.01, ****P* < 0.001, *****P* < 0.0001, ANOVA and Bonferroni multiple comparison test for (E-I).

**Figure S4. (A)** A549 cells were transfected with indicated siRNAs, followed by the poly(I:C) treatment (1μg/mL). Cell samples were processed for ChIP-PCR to determine the H3Ac association at the promoter region of IFN-β. **(B)** A549 cells were transfected with NAT10 or non-targeting siRNAs (1:1 mixture of siNAT10-1 and siNAT10-2, siNT), followed by poly(I:C) treatment. Cell samples were processed for measurement of chromatin accessibility at the promoter region of IFN-β. **(C)** A549 cells were transduced with V5-tagged NAT10 WT or G641E, followed by poly(I:C) treatment (1μg/mL). Cell samples were processed for ChIP-PCR to determine the IRF3 association at the promoter region of IFN-β. **(D)** A549 cells were transfected with indicated siRNAs and treated with poly(I:C) (1μg/mL). Total IRF3 and phospho-IRF3(S386) proteins were analyzed by immunoblotting using their specific antibodies. GAPDH was used as a loading control. **(E)** A549 cells were transfected with the indicated siRNAs and challenged with VSV viruses, the IRF3 protein dimerization were analyzed by immunoblotting using the native PAGE gel. **(F, G)** A549 cells were transfected with the indicated siRNAs, followed by the transient transfection of the vectors expressing IRF3-5D. The IFN-β expression was measured by RT-qPCR (F). IRF3-5D association at the promoter region of IFN-β was measured by ChIP-PCR using an anti-FLAG antibody (G). **(H)** A549 cells were transfected with the indicated siRNAs and treated with poly(I:C) (1μg/mL). Cells samples were processed for ChIP-PCR to determine the IRF3 association at the promoter region of ISG15. **(I)** HEK293T cells were transfected with the indicated siRNAs, followed by co-transfection of the PRDII-luciferase firefly reporter vector, the renilla luciferase vector, and the RIG-I 2CARD vector. The firefly *vs* renilla  luminescence  was quantified, and the relative light units (RLU) were calculated. **(J)** A549 cells were transfected with the indicated siRNAs and treated with poly(I:C) (1μg/mL). The level of lncMxA RNA was measured by RT-qPCR. **(K)** HEK293T cells were transfected with the indicated siRNAs and treated with poly(I:C) (1μg/mL). The total RNAs were harvested at the indicated timepoint post of Actinomycin D treatment (10 μg/ml), and the LncMxA RNA level was measured by RT-qPCR. **(L)** A549 cells were transduced with lentiviral vectors expressing lnc-MxA, and the lncMxA RNA level was measured by RT-qPCR. The results were presented as the Mean ± S.E.M.; ns, not significant, **P* < 0.05, ***P* < 0.01, ****P* < 0.001, *****P* < 0.0001. ANOVA and Bonferroni multiple comparison test for (F,I) or two-tailed Student t-test for (A-C,G,H,J,L).

**Figure S5. (A)** A549 cells were treated with remodelin (5 μM), followed by poly(I:C) treatment. The lncMxA RNA level was measured by RT-qPCR. **(B)** A549 cells were treated with remodelin (5 μM), followed by challenge with influenza viruses. Expression of flu NP was measured by RT-qPCR. **(C)** Supernatants from A549 cells were treated with remodelin at indicated doses, either un-infected or infected with influenza, which were collected to quantify the secreted IFN-β protein by ELISA. The results were presented as the Mean ± S.E.M.; ns, not significant, **P* < 0.05. ANOVA and Bonferroni multiple comparison test for (C) or two-tailed Student t-test for (A,B).
